# 18-Years of single-centre DNA testing in over 7000 index cases with inherited retinal dystrophies and optic neuropathies

**DOI:** 10.1038/s41598-024-77014-4

**Published:** 2024-10-26

**Authors:** Christina Kiel, Fabiola Biasella, Heidi Stöhr, Philipp Rating, Georg Spital, Ulrich Kellner, Karsten Hufendiek, Cord Huchzermeyer, Herbert Jaegle, Klaus Ruether, Bernhard H. F. Weber

**Affiliations:** 1 Institute of Human Genetics, University of Regensburg, Franz-Josef-Strauss-Allee 11, 93053 Regensburg, Germany; 2 Department of Ophthalmology, University Hospital Essen, Hufelandstraße 55, 45147 Essen, Germany; 3 Augenzentrum am St. Franziskus-Hospital, Hohenzollernring 74, 48145 Münster, Germany; 4 Center for Rare Retinal Diseases, AugenZentrum Siegburg, Europaplatz 3, 53721 Siegburg, Germany; 5 RetinaScience, Postfach 301212, 53192 Bonn, Germany; 6 Hannover Medical School, University Clinic of Ophthalmology, Carl-Neuberg-Straße 1, 30625 Hannover, Germany; 7 Department of Ophthalmology, University Hospital Erlangen, Schwabachanlage 6, 91054 Erlangen, Germany; 8 Department of Ophthalmology, University Hospital Regensburg, Franz-Josef-Strauss-Allee 11, 93053 Regensburg, Germany; 9 Specialist Practice Ophthalmology, Dorotheenstraße 56, 10117 Berlin, Germany; 10 Institute of Clinical Human Genetics, University Hospital Regensburg, Franz-Josef-Strauss-Allee 11, 93053 Regensburg, Germany

**Keywords:** Inherited retinal disease, IRD, DNA testing, Next-generation sequencing, Genetic variants, Diagnostic yield, Clinical genetics, Consanguinity, Genotype, Medical genetics, Mutation

## Abstract

Inherited retinal dystrophies (IRDs) and inherited optic neuropathies (IONs) are characterized by distinct genetic causes and molecular mechanisms that can lead to varying degrees of visual impairment. The discovery of pathogenic variants in numerous genes associated with these conditions has deepened our understanding of the molecular pathways that influence both vision and disease manifestation and may ultimately lead to novel therapeutic approaches. Over the past 18 years, our DNA diagnostics unit has been performing genetic testing on patients suspected of having IRD or ION, using state-of-the-art mutation detection technologies that are continuously updated. This report presents a retrospective analysis of genetic data from 6237 IRD and 780 ION patients. Out of these, 3054 IRD patients (49.0%) and 211 ION patients (27.1%) received a definitive molecular diagnosis, with disease-causing variants identified in 139 different genes. The genes most implicated in disease pathologies are *ABCA4*, accounting for 23.8% of all IRD/ION index cases, followed by *BEST1* (7.8%), *USH2A* (6.2%), *PRPH2* (5.7%), *RPGR* (5.6%), *RS1* (5.5%), *OPA1* (4.3%), and *RHO* (3.1%). Our study has compiled the most extensive dataset in combined IRD/ION diagnostics to date and offers valuable insights into the frequencies of mutant alleles and the efficiency of mutation detection in various inherited retinal conditions.

## Introduction

Inherited retinal dystrophies (IRDs) and inherited optic neuropathies (IONs) are genetic disorders that significantly contribute to global blindness. These conditions consist of a diverse group of single-gene disorders, characterized by substantial variability in onset and clinical presentation. Symptoms can appear at any point from birth through late adulthood, often manifesting as increased sensitivity to glare, night and peripheral vision difficulties, decreased central visual acuity, scotomas, or color vision abnormalities. While some forms of IRD and ION are mild and remain stable for many years, most are progressive and may eventually result in legal blindness.

Data on disease prevalence across various populations indicate that IRDs affect approximately 1 in 1340 individuals^1,2^. It is estimated that approximately 5.5 million individuals worldwide are affected by autosomal recessive IRD subtypes alone, with a global carrier frequency of 36% for mutations that cause these diseases^1^. Autosomal dominant optic atrophy (ADOA) and Leber hereditary optic neuropathy (LHON) are the most common IONs with a prevalence of ADOA of 1 in 25,000^3^, while the values for LHON range from 1 in 31,000 to 1 in 68,000^4–7^. Autosomal dominant, X-linked, and mitochondrial-associated IRDs/IONs often exhibit reduced penetrance^8,9^.

IRDs may affect specific cell types, like cone dystrophy, or specific regions, such as macular dystrophy, but often spread across the entire retina as the disease progresses^10^. IONs refer to a spectrum of optic nerve diseases mainly due to ganglion cell degeneration^11^. Additionally, IRDs and IONs can be part of multisystemic diseases, with over 80 syndromic IRD forms identified to date^12^. Both syndromic and non-syndromic diseases arise from pathogenic sequence variations in over 300 genes, each playing a distinct role in the complex processes of light perception (RetNet, the Retinal Information Network, accessed in September 2024). Mutations in these genes can disrupt various cellular processes, including phototransduction, transcription, and mitochondrial function^13^.

The advent of high-throughput next-generation sequencing (NGS) has revolutionized molecular diagnostics for genetically heterogeneous disorders. Previously, diagnosing IRDs/IONs through Sanger sequencing of selected candidate genes and DNA microarray analysis to detect known sequence variants was labor-intensive and yielded limited results^14–16^. In the past decade, targeted multigene panel-based sequencing and whole exome sequencing have become standard practice in IRD/ION genetic diagnostics, significantly enhancing the likelihood of identifying the genetic basis of the disease. This progress is evidenced by an increasing volume of published genetic data from reasonably sized IRD/ION patient cohorts worldwide^8,17–19^. More recently, whole genome sequencing has become increasingly utilized in routine IRD/ION diagnostics^20^, enabling the detection of rare, complex structural and non-coding variants in intronic, intergenic and regulatory regions^21^. The considerable phenotypic overlap between different IRD pathologies and the variable expression within families are well-documented. As a notable example, autosomal dominant IRD associated with variants in the *PRPH2* gene are known to present with a broad clinical presentation including retinitis pigmentosa (RP), different types of macular dystrophy (MD), severe choroidal atrophy as well as asymptomatic carriers even within the same family^22^. The complexity of IRD/ION is further increased by the fact that variants in a gene can cause variable disease phenotypes that follow different modes of inheritance. This is exemplified by the bestrophinopathies, which are linked to variants in the bestrophin-1 (*BEST1*) gene but are inherited in either an autosomal dominant (as seen in Best vitelliform macular dystrophy and vitreoretinochoroidopathy) or in an autosomal recessive manner (as seen in bestrophinopathy, autosomal recessive)^23^. Therefore, multiplex genetic testing is crucial for identifying atypical presentations and non-specific clinical phenotypes.

Gene- or mutation-specific treatment options for various IRD and ION phenotypes, including RP, Leber congenital amaurosis (LCA), choroideremia (CHM), Usher syndrome (USH), X-linked retinoschisis (RS), LHON and Stargardt disease, are currently under evaluation in over 200 clinical trials^24,25^. Following the successful market launch of a subretinal gene augmentation therapy for IRDs caused by biallelic *RPE65* mutations (voretigene neparvovec, Luxturna®, Spark Therapeutics, Inc.), several studies are exploring viral vector-based approaches to deliver functional gene copies for autosomal recessive or X-chromosomal forms of IRDs and IONs. Furthermore, the development of CRISPR/Cas9 gene editing technology has introduced new methods for treating IRDs and IONs, including gene disruption and precise gene correction techniques^26^. For instance, therapies targeting aberrant splicing of the *CEP290* transcript in IRD patients with the deep intronic c.2991 + 1655A > G mutation are being tested both in preclinical and clinical settings^27,28^. Besides improving clinical management and prognosis, a precise genetic diagnosis is increasingly important for IRD/ION patients, particularly in personalizing treatment options and accessing clinical trials.

The profound progress in genetic diagnostics has significantly broadened the knowledge of the mutation spectrum of genes associated with IRD/ION, as demonstrated by the growing repository of putative disease-causing sequence variations in databases such as the Human Gene Mutation Database (HGMD) and ClinVar^29,30^. Our study adds to the growing volume of DNA variation associated with human disease underlying the IRD/ION phenotypes and presents results obtained from an in-depth retrospective analysis of genetic data from over 7000 index patients generated in our single center diagnostics laboratory over a time span of almost two decades.

## Methods

### Cohort and ethical considerations

Most patients suspected of having IRDs/IONs are referred directly to the diagnostic laboratory of the Institute of Human Genetics at the University of Regensburg, Germany, by experienced ophthalmologists from specialist clinics across Germany. Genetic counselling, blood or tissue sampling and DNA testing was done in strict conformity with the applicable rules as stipulated in the German Genetic Diagnostics Act (GenDG). Our internal laboratory information system, GEPADO Xpro (GEPADO GmbH, Dresden, Germany), was used to identify 7017 presumably unrelated patients (referred to as index patients) who underwent genetic testing between January 2006 and July 2023 (a detailed breakdown of the number of patients per year is provided in Supplementary Fig. 1). For 859 index patients at least one family member was available for segregation analysis (total number of relatives is 1546). Participants in this study provided written informed consent for DNA analysis and the sharing of scientific data in accordance with institutional review board guidelines. Ethnicity estimations for the index patients were conducted using the full name model of the R rethnicity package^31^. The study adhered to the principles of the Declaration of Helsinki and was approved by the Ethics Committee of the University of Regensburg (24-3928-104).

IRD/ION index patients were categorized in 1 of 12 subgroups based on the clinical phenotype information provided by the referring specialist physician: achromatopsia (ACHM), Bardet-Biedl syndrome (BBS), cone dystrophy/cone-rod dystrophy (CD/CRD), CHM, congenital stationary night blindness (CSNB), familial exudative vitreoretinopathy (FEVR), MD, optic atrophy/Leber hereditary optic neuropathy (referred to as ION), RS, RP/LCA, USH, and miscellaneous (Supplementary Table 1). The “miscellaneous” category includes patients with suspected Alstrom syndrome, Bietti crystalline corneoretinal dystrophy, Jalili syndrome, and other unspecified retinal diseases (Supplementary Table 1).

### DNA analysis

DNA was extracted from peripheral white blood cells using one of the following methods: the FlexiGene DNA Kit (Qiagen, Hilden, Germany), the MagNA Pure Compact System (Roche, Penzberg, Germany), or the EZ2 Connect System (Qiagen). The quantity of DNA was determined using the NanoDrop One spectrophotometer or the Qubit Fluorometer (ThermoFisher Scientific, Dreieich, Germany). Quality control of the DNA was conducted using the NanoDrop One spectrophotometer, the 2100 Bioanalyzer, or the 4150 TapeStation System (Agilent, Santa Clara, CA, USA).

Molecular genetic techniques were applied based on the technological standards available at the time of patient referral. For some patients who underwent multi-tiered diagnostics over several years, only the results from the most recent tests were used for data analysis. Single or sequential candidate gene testing was conducted on 2317 index patients (33.0%) using PCR followed by Sanger chain-termination sequencing with the ABI3130xl Genetic Analyzer or SeqStudio 4 (Applied Biosystems, Carlsbad, CA, USA). APEX-based microarrays, designed to selectively screen for known sequence variations in several IRD genes (Asper Ophthalmics, Tartu, Estonia), or genotyping using a custom-designed GeneChip CustomSeq Resequencing microarray (Affymetrix, Santa Clara, CA, USA)^32^ was performed on 1031 index patients (14.7%). From 2012 onwards, the majority of the index patients, numbering 3653 (52.1%), were analysed using NGS-based custom-designed multigene panels that comprised a varying number of preselected IRD/ION genes. The composition of these multigene panels was continuously updated based on information accessed via RetNet (https://web.sph.uth.edu/RetNet/) and literature research. The latest version of the panel included 289 IRD/ION-associated genes, covering known deep intronic variants and non-coding regulatory regions of several genes (Supplementary Table 2). Subpanels for individual patients were selected for first-tier testing based on the a priori diagnosis provided by the clinicians. Patients without a positive genetic testing result were screened for variations in all genes included in the panel when consent for the extended analysis was given. Initially, NGS was performed using an Ion Torrent semiconductor personal sequencing machine (ThermoFisher Scientific)^32^. From 2015 onward, reversible-terminator end-to-end sequencing-by-synthesis technology was accomplished on the MiSeq or NextSeq 550 platforms (Illumina, San Diego, CA, USA). Sanger resequencing of the highly repetitive GC-rich exon 15 of the *RPGR* gene (ORF15) was performed for unsolved male cases with suspected IRD subtypes MD, RP or CRD when NGS coverage decreased below 10 × and for all suspected female carriers of X-chromosomal IRD. Screening for copy number variations was carried out by multiplex ligation-dependent probe amplification or by NGS-based in silico tools.

Data from Sanger sequencing, resequencing microarrays, and multiplex ligation-dependent probe amplification was analysed using the Sequence Pilot software (JSI Medical Systems, Kippenheim, Germany). NGS data were analysed using the CLC Genomics Workbench (Qiagen).

### Variant classification

The descriptions of sequence variations identified over the years have been adapted to align with the recommendations of the latest version (21.0) of the Human Genome Variation Society (HGVS) nomenclature. Additionally, all variants were annotated based on a single gene reference sequence (Supplementary Tables 2 and 3). The assessment of pathogenicity for each sequence variation was based on mutation type and information available at the time of analysis including previous reports of the same or similar alterations in other IRD/ION patients, allele frequency with a variant allele frequency cutoff of 1% for all genes analyzed, segregation analysis, functional studies, and bioinformatic prediction software, following the guidelines of the American College of Medical Genetics and Genomics (ACMG)^33^. The classification of sequence alterations was facilitated by updated versions of the Alamut™ variant annotation and interpretation software (SOPHiA Genetics, Bidart, France) and HGMD Professional (Qiagen). For sequence alterations that had been repeatedly detected with differing pathogenicity assessments, the most recent interpretation was accepted for this study. Sequence variants not listed in HGMD Professional version 2023.2 were considered novel. All sequence variants presented in Supplementary Table 3 have been submitted to the ClinVar database.

### Classification of genetic results

Index patients were considered to have a confirmed genetic diagnosis and were classified as a “solved case” if they carried either (1) a monoallelic “pathogenic” or “likely pathogenic” variant in an IRD/ION gene associated with autosomal dominant or X-chromosomal inheritance, or (2) a homozygous “pathogenic” or “likely pathogenic” variant, or two heterozygous “pathogenic” or “likely pathogenic” variants in an IRD/ION gene associated with autosomal recessive inheritance. The latter category also includes patients with a heterozygous “pathogenic” or “likely pathogenic” variant alongside a known “hypomorphic” variant in the same gene. Notable hypomorphic variants include: c.5603A > T in *ABCA4*^32,34^, c.220C > G in *ACO2*^35^, c.1843G > A in *HGSNAT*^36^, and c.5797C > T in *RP1*^37^. Since the allele frequency of the c.5603A > T variant in the *ABCA4* gene is > 1% and was routinely filtered out, patients found to carry one or more (likely) pathogenic variants in the *ABCA4* gene were additionally screened for carriership of this frequent hypomorphic variant**.** Assumed compound heterozygosity of two or more sequence variants in the same gene was analysed whenever segregation analysis in family members was possible, although confirmation of biallelism was not required to classify a case as solved. The *cis* status of common complex alleles in the *ABCA4* gene, such as c.[2588G > C;5603A > T]^17,38^, c.[4469G > A;5603A > T]^17,39^, c.[5461-10 T > C;5603A > T]^17,40^, and c.[1622 T > C;3113C > T]^17,41^, was generally not confirmed. Patients with known LHON-causing mutations in mitochondrial genes (*MT-ND1* m.3460G > A, *MT-ND4* m.11778G > A, *MT-ND6* m.14484 T > C) were also considered as solved cases.

## Results

### Cohort description

Genetic data from a total of 7017 index patients with various types of suspected IRDs/IONs (Supplementary Table 1) were evaluated retrospectively. Of these, 6237 patients were assigned to one of the IRD subgroups, and 780 to the ION group. The IRD patient cohort consisted of nearly equal numbers of males (n = 3262, 52.3%) and females (n = 2975, 47.7%), representing a balanced gender distribution. In X-linked IRDs, such as RS and CHM, the gender ratio was skewed towards males (Table 1). In the ION group, 491 (62.9%) individuals were males, while 289 (37.1%) were females. The ages of the patients at the time of diagnostic testing ranged from under 1 to 90 years, with a mean age of 37.6 (± 19.5) years for IRD cases and 37.2 (± 18.9) years for ION cases (Table 1). The assessment of potential ethnicities based on given names and surnames indicated that half of the cohort likely consists of white individuals, presumably of German ancestry, while the other half is made up of a mix of various ethnicities. More than 65% of the patients in the cohort were affected by either some form of MD (n = 2333; 33.2%) or RP/LCA (n = 2256; 32.2%), while the proportion of other IRD/ION subtypes (excluding miscellaneous) ranged from 1 to 11% (Table 1). Syndromic conditions were largely limited to 286 patients with combined hearing and visual impairments (USH type) and 83 cases with suspected BBS.

**Table 1:** Characterization of the cohort by IRD/ION phenotype, gender and mean age at DNA testing.

IRD subtype	N_total_	N_female_	N_male_	mean age (± SD)
ACHM	69	32 (46.4%)	37 (53.6%)	18.5 (± 16.2)
BBS	83	35 (42.2%)	48 (57.8%)	13.9 (± 10.7)
CD/CRD	645	311 (48.2%)	334 (51.8%)	38.4 (± 19.7)
CSNB	78	28 (35.9%)	50 (64.1%)	25.9 (± 17.8)
CHM	118	35 (29.7%)	83 (70.3%)	42.4 (± 17.9)
FEVR	98	33 (33.7%)	65 (66.3%)	13.3 (± 14.8)
ION	780	289 (37.1%)	491 (62.9%)	37.2 (± 18.9)
MD	2333	1202 (51.5%)	1131 (48.5%)	41.1 (± 17.8)
RP/LCA	2256	1115 (49.4%)	1141 (50.6%)	38.3 (± 19.5)
USH	286	146 (51.0%)	140 (49.0%)	34.6 (± 17.3)
XLRS	232	18 (7.8%)	214 (92.2%)	21.7 (± 17.7)
Miscellaneous	39	20 (51.3%)	19 (48.7%)	38.1 (± 18.3)
Total	7017	3264 (46.5%)	3753 (53.5%)	37.5 (± 19.4)

SD, standard deviation; ACHM, Achromatopsia, BBS, Bardet-Biedl syndrome, CD, Cone dystrophy, CRD, Cone rod dystrophy, CSNB, Congenital stationary night blindness, CHM, Choroideremia, FEVR, familiar exudative vitreoretinopathy, ION, inherited optic neuropathies, MD, Macular dystrophy, RP, Retinitis pigmentosa, LCA, Leber congenital amaurosis, USH, Usher syndrome, XLRS, X-linked retinoschisis.

### Diagnostic rates

**Fig. 1:**
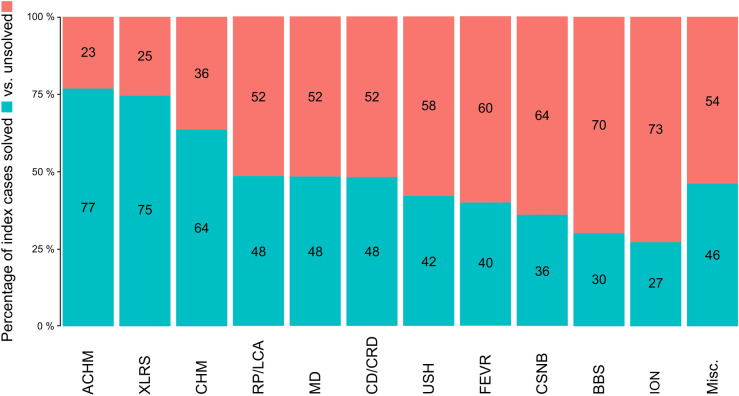
Diagnostic yield per indication group. Shown are the proportions of solved vs unsolved index cases in the different indication groups. A patient was classified as solved case if one (likely) pathogenic variant with dominant, X-linked or mitochondrial inheritance was detected or alternatively when two (likely) pathogenic alleles or one (likely) pathogenic allele and one hypomorphic allele were present in recessive conditions. ACHM, Achromatopsia, XLRS, X-linked retinoschisis, CHM, Choroideremia, RP, Retinitis pigmentosa, LCA, Leber congenital amaurosis, MD, Macular dystrophy, CD, Cone dystrophy, CRD, Cone rod dystrophy, USH, Usher syndrome, FEVR, familiar exudative vitreoretinopathy, CSNB, Congenital stationary night blindness, BBS, Bardet-Biedl syndrome, ION, inherited optic neuropathies, Misc. , Miscellaneous.

Out of the 7017 index patients, a total of 3265 individuals received a definitive molecular genetic diagnosis, resulting in an overall diagnostic rate of 46.5%. Among the IRD group, 3054 out of 6237 cases (49.0%) achieved a definitive molecular diagnosis, whereas in the ION group, 211 out of 780 cases (27.1%) were solved. The “solved case” rate varied considerably across individual IRD subgroups (Fig. 1). A diagnostic yield of more than 60% was observed in patients with clearly discernible phenotypes and little or no genetic heterogeneity, specifically ACHM (76.8%, 53/69 cases solved), X-linked RS (74.6%, 173/232 cases solved), and CHM (63.6%, 75/118 cases solved). Diagnostic yields between 40 and 50% were achieved in patients with RP/LCA (48.3%, 1089/2256 cases solved), MD (48.2%, 1123/2333 cases solved), CD/CRD (48.1%, 310/645 cases solved), and USH (42.3%, 121/286 cases solved). The lowest rates of solved cases in the IRD group were found in patients with FEVR (39.8%, 39/98 cases solved), CSNB (35.9%, 28/78 cases solved), and BBS (30.1%, 25/83 cases solved). Notably, the majority of patients with BBS were analysed using APEX microarrays, likely explaining the intrinsically low diagnostic success rate. Among the 39 patients in the “miscellaneous” subgroup, 18 (46.2%) cases were solved (Fig. 1).

### Testing of family members

In 859 index cases, family members were available for targeted testing and segregation analysis. For 63.2% (n = 543), the information from testing of the relatives was helpful in either supporting or confirming the molecular genetic diagnosis of the index patient (Supplementary Table 3). Briefly, in 251 families (226 IRD and 25 ION), the likely pathogenic or pathogenic variants segregated with the disease in two to five affected family members. The phase could be determined in 325 index patients (321 IRD and 4 ION). Compound heterozygosity was confirmed in 170 patients and supported in additional 82 patients (in case only one relative was available). Homozygosity was confirmed in 55 index patients, de novo variants were identified in 12 individuals. The presence of likely pathogenic or pathogenic variants located on one allele (cis) was found in six index cases, resulting in the correction of the molecular diagnosis for one index patient from ‘solved’ to 'unsolved.

### Inheritance patterns in solved cases

In more than half of the 3054 solved IRD cases (n = 1742, 57.0%), the IRD phenotype was transmitted in an autosomal recessive mode, with 553 individuals (31.7%) carrying homozygous alleles. In 793 solved cases (26.0%), pathogenic sequence variants were inherited in an autosomal dominant manner, while in two cases we found both autosomal dominant and autosomal recessive gene variants to be present in a single patient. X-linked IRD was less common, comprising 515 patients with a confirmed diagnosis (16.9%). Two index patients (0.07%) assigned to the IRD group carried one of the three known pathogenic variants in mitochondrial DNA causing LHON disease.

In the ION group, 146 of the 211 solved patients carried variants consistent with autosomal dominant disease (69.2%), followed by 48 patients with one of the three mitochondrial DNA point mutations known to cause LHON (22.7%). In 14 patients (6.6%) variants confirming autosomal recessive ION were identified, 9 of them carried homozygous alleles (64.3%). X-linked ION was found in 3 individuals (1.4%).

Five probands in our IRD cohort carry two distinct genetic causes that explain their respective retinal phenotypes (Supplementary Table 3). In two simplex RP patients, disease-causing variants compatible with both autosomal dominant and autosomal recessive inheritance of IRD were detected. The first patient, a 49-year-old male (ID 14859), carries two heterozygous sequence variants in the *USH2A* gene (c.2276G > T and c.14849_14858dup) and a heterozygous missense mutation in the *PRPF4* gene (c.944C > T). The c.2276G > T; p.(Cys759Phe) mutation is a known pathogenic *USH2A* variant, while the c.14849_14858dup; p.(Asp4953Glufs*4) variant, although not previously reported, is located in a region of the *USH2A* gene where mutations involving deletion, insertion, or duplication of one or multiple base pairs have previously been reported^42^. The likely pathogenic c.944C > T; p.(Pro315Leu) missense variant in the *PRPF4* gene, initially identified in four members of a Han Chinese family with early- to medium-onset RP, was shown through experimental studies in cellular models to affect *PRPF4* gene expression induced by the amino acid exchange^43^.

The second simplex RP case involves an 18-year-old female (ID 31374) found to carry two heterozygous sequence variants in the *EYS* gene and a heterozygous missense mutation in the *PRPH2* gene. The 6416G > A; p.(Cys2139Tyr) is a frequently identified likely pathogenic *EYS* gene mutation, and the pathogenic c.7228_7228 + 1delinsTA variant at the exon/intron boundary is predicted to disrupt the canonical donor splice site of exon 36 of the *EYS* gene, leading to aberrant mRNA splicing. The latter variant has not been previously reported in the literature, but other splice mutations affecting the donor splice site of exon 36 have been classified as pathogenic^44^. The likely pathogenic c.623G > A; p.(Gly208Asp) missense variant in the *PRPH2* gene is known to cause various forms of autosomal dominant IRD and has been identified in an additional six unrelated individuals in our cohort (Supplementary Table 3).

Three other patients were identified with two plausible distinct genetic causes, two with heterozygous sequence variants in two genes with an autosomal dominant mode of inheritance, and one with homozygous sequence variants in two genes with an autosomal recessive mode of inheritance. A 35-year-old male (ID 9039) with clinical signs of RP was found to carry a known likely pathogenic c.658C > T; p.(Arg220Trp) missense variant in the *PRPH2* gene and a likely pathogenic c.854C > G; p.(Pro285Arg) missense variant in the *RHO* gene. The latter has been identified in two additional unrelated individuals in our cohort but has not yet been reported in the literature. Moreover, a 46-year-old patient (ID 8289) with cone-rod dystrophy, whose great uncle may also have been affected, was found to harbour a known likely pathogenic c.122G > C; p.(Arg41Pro) missense variant in the *CRX* gene and a likely pathogenic c.527 T > C; p.(Leu176Pro) missense variant in the *GUCA1A* gene. The latter variant has not been reported before, but another sequence change affecting codon 176 of the *GUCA1A* gene, c.526C > T; p.(Leu176Phe), has previously been associated with MD^45,46^.

Finally, a 36-year-old male patient (ID 28183) with RP from a consanguineous marriage is a homozygous carrier of known IRD-causing variants in the *RDH5* [c.632_633del; p.(Pro211Argfs*47)] and *WDR19* [c.2777G > T; p.(Ser926Ile)] genes. As additional family members were unwilling to cooperate, further studies (e.g., segregation analysis) were not performed.

### Mutation spectrum and variant analysis

Disease-causing variations in solved cases were identified in 133 genes for the IRD group and in 13 different genes for the ION group (Supplementary Table 4). Variants in seven genes were found to be disease-causing in both groups, namely *CACNA1F*, *GUCY2D*, *MT-ND4*, *MT-ND6*, *OPA1*, *RP1L1* and *WFS1*. Overall, disease-causing variants in 26 genes were identified in only one individual of our 3,265 solved cases with IRD or ION.

**Fig. 2:**
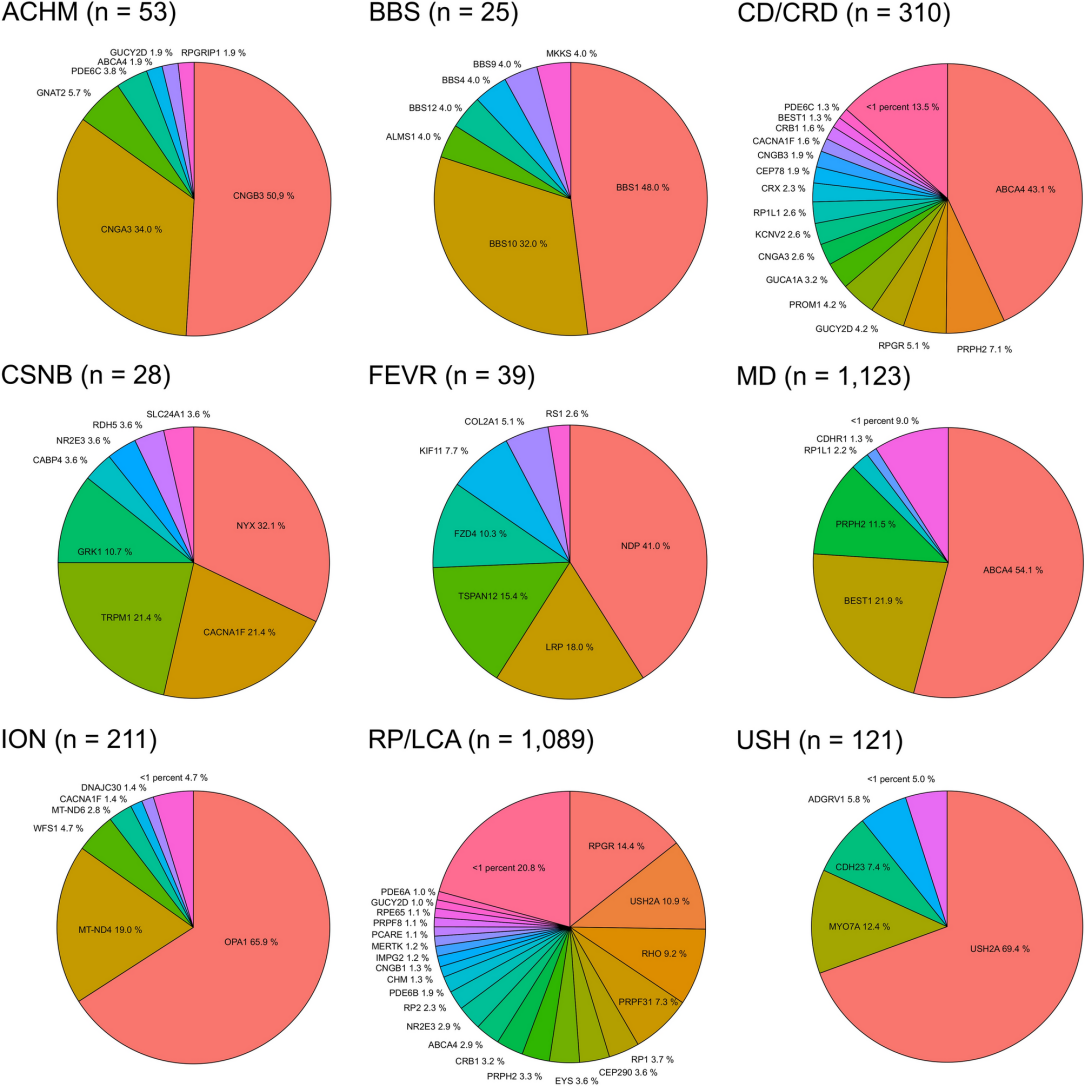
Causative genes identified in solved patients by indication group. Shown are the causative genes identified in 3265 solved patients. In 5 patients more than one potential causative gene was identified, respectively in four patients with RP and one patient with CD/CRD. In these cases, both potential causative genes were considered. Only indication groups with more than one potential causative gene are shown, the indication groups X-linked retinoschisis and choroideremia are expected to be solved by each one gene, namely *RS1* and *CHM*, and are therefore not displayed. Total numbers how often a gene was identified to be causative in the overall cohort and in each indication group are given in Supplementary Table 4. N = number of solved patients in each group.

Almost two-thirds of the 3054 solved IRD cases (n = 1887, 61.8%) were attributed to causative mutations in seven genes, each responsible for affecting more than 100 IRD patients. The most frequently associated IRD gene in our cohort is *ABCA4*, affecting 25.4% of the solved IRD cases (777/3,054), followed by *BEST1* (8.4%, 255/3,054), *USH2A* (6.7%, 204/3,054), *PRPH2* (6.1%, 187/3,054), *RPGR* (6.0%, 183/3054), *RS1* (5.9%, 181/3,054), and *RHO* (3.3%, 101/3,054). A total of 126 IRD genes were linked to 1142 IRD patients, with disease-causing variants in each of these genes ranging between one to 88 individuals (Supplementary Table 4). Note that for the five patients with two potentially causative genes described above, both genes were considered leading to discrepant numbers in patient counts (Supplementary Table 4). Except for the RP/LCA group, in each of the remaining 10 IRD subgroups, sequence changes in one to four genes were responsible for at least half of the patient’s phenotypes (Fig. 2, Supplementary Table 4). In the RP/LCA group, the seven most commonly mutated genes collectively exceed 50%.

Among the 211 ION cases with a confirmed molecular diagnosis, the vast majority, namely 179 (84.8%), carried pathogenic alterations either in the *OPA1* (n = 139, 65.9%) gene, or were carriers of the *MT-ND4* mutation (n = 40,19.0%). The remaining twenty-two solved ION patients (8.5%) can be explained by causative variations in another 11 genes (Fig. 2, Supplementary Table 4).

A total of 30 disease-causing alleles were most frequently observed in our solved cases, as defined by an allele count of at least 15 (Table 2). The most common IRD-causing alleles include thirteen sequence changes in the *ABCA4* gene. Furthermore, the top five spots of disease-causing alleles in the entire cohort were occupied by *ABCA4* variants, with the two most frequent alleles being the mild c.5882G > T variant and the common hypomorphic variant c.5603A > T. Other *ABCA4* variants found more than 15 times in our cohort included the complex alleles c.1622 T > C(;)3113C > T (third most frequent allele), c.2588G > C(;)5603A > T (ninth most frequent allele), and c.5461-10 T > C(;)5603A > T (sixteenth most frequent allele). Of all 7017 index cases analysed, 234 (3.3%) carried at least one complex *ABCA4* allele. Splice mutations, as well as frameshift, stop, and missense mutations in the *ABCA4* gene, were also found in the list of the most frequent pathogenic alleles in our study group (Table 2). Additionally, three alleles with mutations in either the *BEST1* or *USH2A* gene, as well as single disease-causing alleles in the *BBS1*, *CDHR1*, *CEP290*, *CNGB3*, *FAM161A*, *RP1L1*, *RPGR*, *RHO*, and *RS1* genes, were identified at least 15 times in the combined IRD/ION cohort (Table 2).

**Table 2:** The most common genetic variants identified in solved cases of the IRD/ION cohort.

Gene	cPos	pPos	Allele count	Allele fraction (per gene) (%)
*ABCA4* (NM_000350.3)	c.5882G > A	p.(Gly1961Glu)	205	12.1
*ABCA4* (NM_000350.3)	c.5603A > T	p.(Asn1868Ile)	158	9.4
*ABCA4* (NM_000350.3)	c.1622 T > C(;)3113C > T^§^	p.(Leu541Pro)(;) (Ala1038Val)	130	7.7
*ABCA4* (NM_000350.3)	c.5917del	p.(Val1973*)	84	5
*ABCA4* (NM_000350.3)	c.5714 + 5G > A	p.(Glu1863Leufs*33)	61	3.6
*USH2A* (NM_206933.4)	c.2299del	p.(Glu767Serfs*21)	53	12.5
*ABCA4* (NM_000350.3)	c.5461-10 T > C	p.[Thr1821Aspfs*6, Thr1821Valfs*13]	49	2.9
*CNGB3* (NM_019098.5)	c.1148del	p.(Thr383Ilefs*13)	47	61.8
*ABCA4* (NM_000350.3)	c.2588G > C(;)5603A > T^§^	p.[Gly863Ala,Gly863del] (;)(Asn1868Ile)	42	2.5
*MT-ND4* (NC_012920.1)	c.1019G > A	p.(Arg340His)	41	100
*USH2A* (NM_206933.4)	c.11864G > A	p.(Trp3955*)	40	9.5
*BEST1* (NM_004183.4)	c.728C > T	p.(Ala243Val)	38	12.3
*CEP290* (NM_025114.4)	c.2991 + 1655A > G	p.(Cys998*)	34	40
*USH2A* (NM_206933.4)	c.2276G > T	p.(Cys759Phe)	34	8
*ABCA4* (NM_000350.3)	c.4234C > T	p.(Gln1412*)	33	2
*ABCA4* (NM_000350.3)	c.5461-10 T > C(;) 5603A > T^§^	p.[Thr1821Aspfs*6, Thr1821Valfs*13](;) (Asn1868Ile)	31	1.8
*RP1L1* (NM_178857.6)	c.133C > T	p.(Arg45Trp)	31	70.5
*BEST1* (NM_004183.4)	c.884_886del	p.(Ile295del)	29	9.4
*CDHR1* (NM_033100.4)	c.783G > A	p.[= ,Asp214_Pro261del]	27	71.1
*OPA1* (NM_130837.3)	c.2873_2876del	p.(Val958Glyfs*3)	27	18.6
*ABCA4* (NM_000350.3)	c.4253 + 43G > A	p.[= ,Ile1377Hisfs*3]	24	1.4
*BBS1* (NM_024649.5)	c.1169 T > G	p.(Met390Arg)	24	60
*ABCA4* (NM_000350.3)	c.3113C > T	p.(Ala1038Val)	22	1.3
*RS1* (NM_000330.4)	c.214G > A	p.(Glu72Lys)	20	10.9
*BEST1* (NM_004183.4)	c.422G > A	p.(Arg141His)	18	5.8
*RPGR* (NM_001034853.2)	c.2405_2406del	p.(Glu802Glyfs*32)	17	9.2
*ABCA4* (NM_000350.3)	c.768G > T	p.[= , Leu257Valfs*17]	16	0.9
*ABCA4* (NM_000350.3)	c.6229C > T	p.(Arg2077Trp)	15	0.9
*FAM161A* (NM_001201543.2)	c.1309A > T	p.(Arg437*)	15	83.3
*RHO* (NM_000539.3)	c.1040C > T	p.(Pro347Leu)	15	14.9

Allele frequencies were determined in our cohort of 3265 solved cases. Displayed are all variants with an allele count of at least 15.

^§^alleles were detected in the same individual, the phase was not determined.

The alleles most often identified in ION patients were the LHON-associated c.1019G > A sequence change in the mitochondrial *MT-ND4* gene and a 4-bp deletion (c.2873_2876del) in the *OPA1* gene (Table 2).

In total, 5254 different genetic variants were identified in the index cases, including 3236 novel sequence changes (61.6%) not listed in HGMD (Supplementary Table 3). Of the novel variants, 2279 (70.4%) were classified as variants of unknown significance, which have not been considered as potentially disease-causing in this study. Approximately one-third of the novel variants (904/3236 or 27.9%) were classified as pathogenic or likely pathogenic and mainly constitute nonsense and frameshift mutations (488/904, 54.0%), followed by missense mutations (226/904, 25.0%), splice mutations (124/904, 13.7%), deletions or duplications affecting one or several amino acids or del/dup variations encompassing an entire exon or even several exons (60/904, 6.6%), insertion/deletion variants (5/904, 0.6%), and a single variant predicted to lead to a loss of the start codon ATG.

## Discussion

The current study provides comprehensive data on genetic analyses conducted in over 7000 index patients with various types of IRD and ION that were subjected to molecular diagnosis between January 2006 and July 2023 at the Institute of Human Genetics Regensburg. The index patients were categorized into 11 IRD sub-cohorts according to their medical indication, with each of these sub-cohorts comprising between 39 and 2333 patients and 780 ION cases. Our study represents one of the largest datasets reported so far in the field of molecular diagnostics for ophthalmogenetic disorders. Our findings in 7017 index patients cover a total of 2436 pathogenic or likely pathogenic variants, of which 904 (pathogenic or likely pathogenic) have not been listed in HGMD so far.

A definite molecular diagnosis was achieved in 3265 IRD/ION patients with genetic variants classified as pathogenic or likely pathogenic. This results in an overall diagnostic yield of 46.5%. In line with previous cohort studies^8,18,19,47^, we observed that a core set of 8 genes accounts for over 60% of disease-causing variants, while most known IRD/ION genes contribute only little to disease aetiology. For solved cases genetic variation was characterized as pathogenic (n = 3966 alleles), likely pathogenic (n = 1398 alleles) or hypomorphic (n = 244 alleles), the latter category was only considered if it has contributed to a solved case in combination with a second mutation. The top 3 most recurrent genetic variations were found in *ABCA4* (n = 870 alleles in 13 variants), *USH2A* (n = 127 alleles in 3 variants) and *BEST1* (n = 85 alleles in 3 variants).

Nevertheless, a tiered approach with a stepwise analysis of common and then rare IRD/ION genes seems no longer adequate in the era of cost-effective and time-efficient massively parallel sequencing techniques and highly sophisticated diagnostics pipelines incorporating exome and genome sequencing data. Specifically, the latter two sequencing platforms are increasingly pivotal for the undirected identification of genetic causes of atypical IRD/ION phenotypes, new IRD/ION genes, variants in non-coding genomic regions, and gross structural rearrangements, parameters which recent research has shown to play a significant role in IRD/ION causality^20,32,48–50^.

A recent review and meta-analysis of 105 publications from 28 countries using solely NGS-based methods revealed diagnostic yields in multiple IRD phenotypes ranging from 21 to 100% across all studies, with pooled diagnostic yield estimates between 52 and 74%^51^. In comparison, our diagnostic rate for IRD of 49% is in the lower mid-range of these published values and slightly below the lower limit of the pooled molecular diagnostic yield range. One of the factors that most likely may have reduced our solve rate was the earlier use of mutation detection technologies with lower sensitivity for conducting molecular diagnoses in 3653/7017 of our index patients (e.g. APEX-based microarray analysis). Consistent with this, is a population-based study from Norway using a mix of APEX and NGS panel technologies in IRD diagnostics, that reported a diagnostic yield of only 32%^52^. Some NGS platforms like the Ion Torrent semiconductor technology which we used between 2012 and 2015, is prone to inaccuracies in base calling compared to Sanger sequencing or the sequencing-by-synthesis principle, especially in homopolymeric regions of the genome. Other more general limitations of short-read NGS include coverage gaps and the reduced ability to detect large structural variants or variants in repetitive regions like the ORF15 of the *RPGR* gene. Repeated reanalysis of unsolved cases with modern and optimized sequencing strategies has been conducted consistently over the course of the time period covered in this study to reduce missed diagnoses as best as possible. Time and cost-intensive serial testing will largely become obsolete by introducing long-read genome sequencing technology into routine diagnostics that is known to reliably detect complex structural variants and to resolve challenging regions of the genome (e.g. pseudogenes with almost complete sequence homology to the functional gene).

One of the study-level factors that were reported to be highly variable in different DNA testing reports and significantly influencing the diagnostic rate, was the definition of a solved case with over 60% of the studies failing to provide precise criteria^51^. In this study, we adopted a strict and rather conservative strategy to categorize cases as “solved”, considering only sequence variants classified as pathogenic or likely pathogenic that were consistent with the known gene-associated disease inheritance pattern. This contrasts with most other studies, which considered variants of unknown significance as “potentially causative” for disease in the patients. These probands were commonly referred to as “possibly or likely solved cases” and were included in the calculations of a diagnostic rate^8,53^.

Another parameter known to significantly affect the success rate of molecular diagnosis is the accuracy with which the phenotype of the patient is reported by the referring physician^51,54^. Patients in our cohort were clinically examined at multiple ophthalmology clinics, and some were also seen by clinical geneticists. It can be assumed that the clinical criteria used for diagnosing varied to some extent among individual physicians who referred patients to our diagnostic laboratory, thus introducing a potential bias that may have influenced our diagnostic yield. Interdisciplinary teamwork to integrate clinical, familial, and genetic data would surely be advantageous to optimize and accelerate the process of accurate diagnosis in many cases.

The retrospective nature of our study entails the risk of false interpretation of identified variants due to the previously restricted availability of reliable allele frequencies, genotype data of other IRD/ION patients and controls, and high-performing computing tools to predict pathogenicity. However, potential misinterpretation of the pathogenicity of sequence changes in the earlier years appears no major factor in distorting the success rate of DNA testing in our cohort. For instance, the diagnostic yield for 1401 cases analyzed since 2021, a period during which comprehensive gene panel analyses were exclusively conducted using NGS, was 53.2% (745/1401), thus slightly above the calculated rate for the entire IRD cohort.

The assessment of the pathogenic and likely pathogenic variants identified in our study showed that 90.7% of the variants (2209 of 2436) were found in no more than 3 index patients. In fact, 72.2% of the variants (1759) were mutations found in only a single index patient. This once again highlights the importance of large-scale cohort studies and the public availability of disease-associated genetic variants as the classification of reported genetic variants is a crucial guide for other diagnostic laboratories involved in DNA diagnostics of rare disorders.

Another noticeable finding of our study is the fact that approximately one third (562 out of 1756) of the patients with a recessive inheritance were carrying a homozygous allele as the cause of disease. This relates to 17.2% of all solved cases. Similar numbers were obtained in a large Italian cohort of IRD patients, where the prevalence of homozygous genotypes among recessive clinical phenotypes was 31.2%, resulting in 22.4% of solved cases being homozygous^8^. In this and our study, these numbers appear to reflect a high degree of consanguinity.

In conclusion, our comprehensive dataset which includes the identification of 904 novel, previously unreported (likely) pathogenic variants, represents a valuable resource for elucidating the genetic epidemiology of IRDs and IONs and may aid in the consensual interpretation of extremely rare sequence variants. This will become increasingly important in light of emerging novel gene-specific therapies.

## Supplementary Information

Supplementary Information 1.

Supplementary Information 2.

Supplementary Information 3.

## Data Availability

The entirety of data analysed in this study has been incorporated within the published article and its Supplementary Information file. A total of 5254 variants identified in this study have been submitted to ClinVar (https://www.ncbi.nlm.nih.gov/clinvar/) under accession numbers SCV005068380 – SCV005073654 to ensure community-wide access.
