# Supplementary material for: 18-Years of single-centre DNA testing in over 7000 index cases with inherited retinal dystrophies and optic neuropathies
Source: Sci Rep. 2024 Oct 26;14:25529. doi: 10.1038/s41598-024-77014-4 (PMC11513943; doi:10.1038/s41598-024-77014-4)
Supplement: Supplementary file 1 — Supplementary Information 1. [file 41598_2024_77014_MOESM1_ESM.docx]

Supplementary Information

**18-years of single-centre DNA testing in over 7,000 index cases with inherited retinal dystrophies**

Christina Kiel, Fabiola Biasella, Heidi Stöhr, Philipp Rating, Georg Spital, Ulrich Kellner, Karsten Hufendiek, Cord Huchzermeyer, Herbert Jaegle, Klaus Ruether, Bernhard H.F. Weber

Supplementary Online Material:

Supplementary Figure 1

Supplementary Tables 1-4

**Supplementary Fig. 1.** **Number of patients analysed per year**. The barplot indicates the number of patients who were analysed in the respective year. In case a patient was analysed more than once only the most recent investigation was considered. Data collection was completed at the end of July 2023 and only patients who had been fully analysed by this time were included, therefore the year 2023 does not represent a complete annual summary.


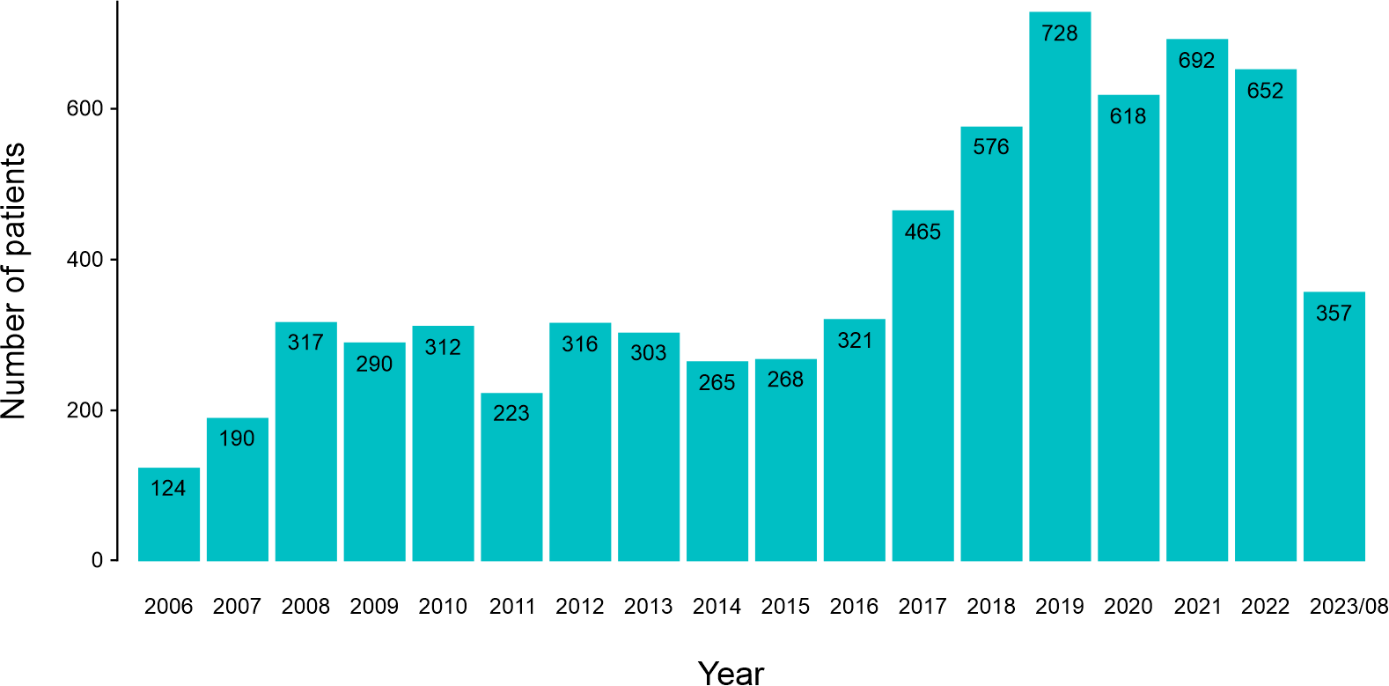


**Supplementary Table 1. Types of inherited retinal disorders.** Clinical indications of patients were defined by the responsible ophthalmologist. Disorders were group by similarity of phenotypes for further investigations.

| **Type of inherited retinal disorder** | **Subtypes (if applicable)** |
| --- | --- |
| **Achromatopsia** | - |
| **Bardet-Biedl syndrome** | - |
| **Cone dystrophy / cone-rod dystrophy** | **cone dystrophy with supernormal rod electroretinogram** |
| **Choroideremia** | - |
| **Congenital stationary night blindness** | - |
| **Familiar exudative vitreoretinopathy** | Norrie disease |
| **Macular dystrophy** | Bestrophinopathy |
|  | Choroidal dystrophy, central areolar |
|  | Doyne honeycomb retinal dystrophy |
|  | Macular dystrophy, patterned |
|  | Macular dystrophy, vitelliform |
|  | Macular dystrophy with hypotrichosis |
|  | North Carolina macular dystrophy |
|  | Occult macular dystrophy |
|  | Sorsby fundus dystrophy |
|  | Stargardt disease |
|  | Vitreoretinochoroidopathy |
| **Miscellaneous** | Alstrom syndrome |
|  | Bietti crystalline corneoretinal dystrophy |
|  | Jalili syndrome |
|  | Unclassified retinal disease |
| **Retinoschisis** | - |
| **Retinitis pigmentosa / Leber congenital amaurosis** | Atrophia gyrata |
|  | Fundus albipunctatus |
|  | Tapetoretinal degeneration |
| **Usher syndrome** | - |

**Supplementary Table 2. List of the 289 IRD-associated genes comprising the in-house panel.** List of the latest version of the gene panel used for IRD diagnostic genetic testing. Included are 289 IRD-associated genes, covering known deep intronic variants and non-coding regulatory regions. For each gene, a direct hyperlink to its description in Mendelian Inheritance in Man (OMIM) is provided (Online Mendelian Inheritance in Man, OMIM®. McKusick-Nathans Institute of Genetic Medicine, Johns Hopkins University, Baltimore, MD. Accessed on 03 April 2024).

| **Gene** | **NM_number** | **Non-coding variants in intronic, intergenic and regulatory regions** | **Genomic position (hg38)** | **Link to Gene / Phenotype description in OMIM** |
| --- | --- | --- | --- | --- |
| *ABCA4* | NM_000350 | c.161-23T>G c.1937+37C>G  c.1937+435C>G  c.1938-514A>G  c.1938-619A>G  c.1938-621G>A  c.2588-706C>T  c.2919-826T>A  c.3050+370C>T  c.3863-1064A>G  c.4253+43G>A  c.4352+61G>A  c.4539+1100A>G  c.4539+1106C>T  c.4539+2001G>A  c.4539+2028C>T  c.4539+2064C>T  c.4539+2065C>G  c.4634+741A>G  c.5196+1137G>A  c.5196+1216C>A  c.5197-557G>T  c.570+1798A>G  c.6148-84A>T  c.6283-78G>T  c.769-784C>T  c.769-788A>T  c.859-506G>C  c.859-540C>G  c.859-546G>A  c.859-640A>G  c.67-2023T>G  c.5196+1056A>G | chr1: 94111602  chr1: 94062540  chr1: 94062142  chr1: 94061273  chr1: 94061378  chr1: 94061380  chr1: 94052404  chr1: 94045570  chr1: 94044243  chr1: 94033107  chr1: 94030953  chr1: 94030367  chr1: 94028345  chr1: 94028339  chr1: 94027444  chr1: 94027417  chr1: 94027381  chr1: 94027380  chr1: 94024213  chr1: 94018445  chr1: 94018366  chr1: 94016411  chr1: 94101217  chr1: 94002076  chr1: 94001183  chr1: 94084225  chr1: 94084229  chr1: 94081224  chr1: 94081258  chr1: 94081264  chr1: 94081358  chr1: 94115089  chr1: 94018526 | <https://omim.org/entry/601691> |
| *ABCC6* | NM_001171 |  |  | <https://omim.org/entry/603234> |
| *ABHD12* | NM_001042472 |  |  | <https://omim.org/entry/613599> |
| *ACBD5* | NM_145698 |  |  | <https://omim.org/entry/616618> |
| *ACO2* | NM_001098 |  |  | <https://omim.org/entry/100850> |
| *ADAM9* | NM_003816 |  |  | <https://omim.org/entry/602713> |
| *ADAMTS18* | NM_199355 |  |  | <https://omim.org/entry/607512> |
| *ADGRA3* | NM_145290 |  |  | <https://omim.org/entry/612303> |
| *ADGRV1* | NM_032119 |  |  | <https://omim.org/entry/602851> |
| *ADIPOR1* | NM_015999 |  |  | <https://omim.org/entry/607945> |
| *AFG3L2* | NM_006796 |  |  | <https://omim.org/entry/604581> |
| *AGBL5* | NM_021831 |  |  | <https://omim.org/entry/615900> |
| *AHI1* | NM_017651 |  |  | <https://omim.org/entry/608894> |
| *AHR* | NM_001621 |  |  | <https://omim.org/entry/600253> |
| *AIPL1* | NM_014336 |  |  | <https://omim.org/entry/604392> |
| *ALMS1* | NM_001378454 |  |  | <https://omim.org/entry/606844> |
| *AP5Z1* | NM_014855 |  |  | <https://omim.org/entry/613653> |
| *ARHGEF18* | NM_001130955 |  |  | <https://omim.org/entry/616432> |
| *ARL2BP* | NM_012106 |  |  | <https://omim.org/entry/615407> |
| *ARL3* | NM_004311 |  |  | <https://omim.org/entry/604695> |
| *ARL6* | NM_001278293 |  |  | <https://omim.org/entry/608845> |
| *ARSG* | NM_014960 |  |  | <https://omim.org/entry/610008> |
| *ASRGL1* | NM_001083926 |  |  | <https://omim.org/entry/609212> |
| *ATF6* | NM_007348 |  |  | <https://omim.org/entry/605537> |
| *ATXN7* | NM_000333 |  |  | <https://omim.org/entry/607640> |
| *BBIP1* | NM_001195306 |  |  | <https://omim.org/entry/613605> |
| *BBS1* | NM_024649 |  |  | <https://omim.org/entry/209901> |
| *BBS10* | NM_024685 |  |  | <https://omim.org/entry/610148> |
| *BBS12* | NM_152618 |  |  | <https://omim.org/entry/610683> |
| *BBS2* | NM_031885 |  |  | <https://omim.org/entry/606151> |
| *BBS4* | NM_033028 |  |  | <https://omim.org/entry/600374> |
| *BBS5* | NM_152384 |  |  | <https://omim.org/entry/603650> |
| *BBS7* | NM_176824 |  |  | <https://omim.org/entry/607590> |
| *BBS9* | NM_198428 |  |  | <https://omim.org/entry/607968> |
| *BEST1* | NM_004183 |  |  | <https://omim.org/entry/607854> |
| *C1QTNF5* | NM_015645 |  |  | <https://omim.org/entry/608752> |
| *C3* | NM_000064 |  |  | <https://omim.org/entry/120700> |
| *CABP4* | NM_145200 |  |  | <https://omim.org/entry/608965> |
| *CACNA1F* | NM_005183 |  |  | <https://omim.org/entry/300110> |
| *CACNA2D4* | NM_172364 |  |  | <https://omim.org/entry/608171> |
| *CAPN5* | NM_004055 |  |  | <https://omim.org/entry/602537> |
| *CC2D2A* | NM_001080522 |  |  | <https://omim.org/entry/612013> |
| *CCDC51* | NM_001256964 |  |  | <https://omim.org/entry/618585> |
| *CCT2* | NM_006431 |  |  | <https://omim.org/entry/605139> |
| *CDH23* | NM_022124 |  |  | <https://omim.org/entry/605516> |
| *CDH3* | NM_001793 |  |  | <https://omim.org/entry/114021> |
| *CDHR1* | NM_033100 NM_001171971 |  |  | <https://omim.org/entry/609502> |
| *CEP164* | NM_014956 |  |  | <https://omim.org/entry/614848> |
| *CEP19* | NM_032898 |  |  | <https://omim.org/entry/615586> |
| *CEP250* | NM_007186 |  |  | <https://omim.org/entry/609689> |
| *CEP290* | NM_025114 |  |  | <https://omim.org/entry/610142> |
| *CEP78* | NM_001098802 |  |  | <https://omim.org/entry/617110> |
| *CERKL* | NM_001030311 |  |  | <https://omim.org/entry/608381> |
| *CFAP410* | NM_004928 |  |  | <https://omim.org/entry/603191> |
| *CFAP418* | NM_177965 |  |  | <https://omim.org/entry/614477> |
| *CFH* | NM_000186 |  |  | <https://omim.org/entry/134370> |
| *CHM* | NM_000390 | c.315-4587T>A  c.315-1536A>G  c.-98C>A  c.-98C>T | chrX: 85968639  chrX: 85965588  chrX: 86047629  chrX: 86047629 | <https://omim.org/entry/300390> |
| *CIB2* | NM_006383 |  |  | <https://omim.org/entry/605564> |
| *CISD2* | NM_001008388 |  |  | <https://omim.org/entry/611507> |
| *CLCC1* | NM_001048210 |  |  | <https://omim.org/entry/617539> |
| *CLN3* | NM_001042432 |  |  | <https://omim.org/entry/607042> |
| *CLRN1* | NM_174878 | c.254-649T>G | chr3:150942410 | <https://omim.org/entry/606397> |
| *CLUAP1* | NM_015041 |  |  | <https://omim.org/entry/616787> |
| *CNGA1* | NM_000087 |  |  | <https://omim.org/entry/123825> |
| *CNGA3* | NM_001298 |  |  | <https://omim.org/entry/600053> |
| *CNGB1* | NM_001297 |  |  | <https://omim.org/entry/600724> |
| *CNGB3* | NM_019098 | c.1663-1205G>A  c.1663-2137C>T  c.212-3599T>A | chr8: 86605416  chr8: 86606348  chr8: 86730256 | <https://omim.org/entry/605080> |
| *CNNM4* | NM_020184 |  |  | <https://omim.org/entry/607805> |
| *COL11A1* | NM_001854 |  |  | <https://omim.org/entry/120280> |
| *COL2A1* | NM_001844 | c.1527+135G>A | chr12: 47986201 | <https://omim.org/entry/120140> |
| *COL9A1* | NM_001851 |  |  | <https://omim.org/entry/120210> |
| *CRB1* | NM_201253 |  |  | <https://omim.org/entry/604210> |
| *CRX* | NM_000554 |  |  | <https://omim.org/entry/602225> |
| *CSPP1* | NM_024790 |  |  | <https://omim.org/entry/611654> |
| *CTNNA1* | NM_001903 |  |  | <https://omim.org/entry/116805> |
| *CTNNB1* | NM_001904 |  |  | <https://omim.org/entry/116806> |
| *CWC27* | NM_005869 |  |  | <https://omim.org/entry/617170> |
| *CYP4V2* | NM_207352 |  |  | <https://omim.org/entry/608614> |
| *DHDDS* | NM_205861 |  |  | <https://omim.org/entry/608172> |
| *DHX38* | NM_014003 |  |  | <https://omim.org/entry/605584> |
| *DNAJC30* | NM_032317 |  |  | <https://omim.org/entry/618202> |
| *DNM1L* | NM_012062 |  |  | <https://omim.org/entry/603850> |
| *DRAM2* | NM_178454 |  |  | <https://omim.org/entry/613360> |
| *DTHD1* | NM_001136536 |  |  | <https://omim.org/entry/616979> |
| *DYNC2H1* | NM_001080463 |  |  | <https://omim.org/entry/603297> |
| *DYNC2I2* | NM_052844 |  |  | <https://omim.org/entry/613363> |
| *EFEMP1* | NM_001039348 |  |  | <https://omim.org/entry/601548> |
| *ELOVL1* | NM_022821 |  |  | <https://omim.org/entry/611813> |
| *ELOVL4* | NM_022726 |  |  | <https://omim.org/entry/605512> |
| *EMC1* | NM_015047 |  |  | <https://omim.org/entry/616846> |
| *ENSA* | NM_004436 |  |  | <https://omim.org/entry/603061> |
| *ESPN* | NM_031475 |  |  | <https://omim.org/entry/606351> |
| *EXOSC2* | NM_014285 |  |  | <https://omim.org/entry/602238> |
| *EYS* | NM_001142800 |  |  | <https://omim.org/entry/612424> |
| *FAM161A* | NM_001201543 |  |  | <https://omim.org/entry/613596> |
| *FBLN5* | NM_006329 |  |  | <https://omim.org/entry/604580> |
| *FDXR* | NM_024417 |  |  | <https://omim.org/entry/103270> |
| *FLVCR1* | NM_014053 |  |  | <https://omim.org/entry/609144> |
| *FZD4* | NM_012193 |  |  | <https://omim.org/entry/604579> |
| *GDF6* | NM_001001557 |  |  | <https://omim.org/entry/601147> |
| *GNAT1* | NM_144499 |  |  | <https://omim.org/entry/139330> |
| *GNAT2* | NM_005272 |  |  | <https://omim.org/entry/139340> |
| *GNB3* | NM_002075 |  |  | <https://omim.org/entry/139130> |
| *GNPTG* | NM_032520 |  |  | <https://omim.org/entry/607838> |
| *GPR179* | NM_001004334 |  |  | <https://omim.org/entry/614515> |
| *GRK1* | NM_002929 |  |  | <https://omim.org/entry/180381> |
| *GRM6* | NM_000843 |  |  | <https://omim.org/entry/604096> |
| *GUCA1A* | NM_000409 |  |  | <https://omim.org/entry/600364> |
| *GUCA1B* | NM_002098 |  |  | <https://omim.org/entry/602275> |
| *GUCY2D* | NM_000180 | c.1378+151C>G | chr17: 8006865 | <https://omim.org/entry/600179> |
| *HADHA* | NM_000182 |  |  | <https://omim.org/entry/600890> |
| *HARS1* | NM_002109 |  |  | <https://omim.org/entry/142810> |
| *HARS2* | NM_012208 |  |  | <https://omim.org/entry/600783> |
| *HGSNAT* | NM_152419 |  |  | <https://omim.org/entry/610453> |
| *HK1* | NM_000188 |  |  | <https://omim.org/entry/142600> |
| *HMX1* | NM_018942 |  |  | <https://omim.org/entry/142992> |
| *IDH3A* | NM_005530 |  |  | <https://omim.org/entry/601149> |
| *IDH3B* | NM_006899 |  |  | <https://omim.org/entry/604526> |
| *IFT140* | NM_014714 |  |  | <https://omim.org/entry/614620> |
| *IFT172* | NM_015662 |  |  | <https://omim.org/entry/607386> |
| *IFT27* | NM_006860 |  |  | <https://omim.org/entry/615870> |
| *IFT74* | NM_025103 |  |  | <https://omim.org/entry/608040> |
| *IFT81* | NM_014055 |  |  | <https://omim.org/entry/605489> |
| *IMPDH1* | NM_000883 |  |  | <https://omim.org/entry/146690> |
| *IMPG1* | NM_001563 |  |  | <https://omim.org/entry/602870> |
| *IMPG2* | NM_016247 |  |  | <https://omim.org/entry/607056> |
| *INPP5E* | NM_019892 |  |  | <https://omim.org/entry/613037> |
| *INVS* | NM_014425 |  |  | <https://omim.org/entry/243305> |
| *IQCB1* | NM_001023570 |  |  | <https://omim.org/entry/609237> |
| *ITM2B* | NM_021999 |  |  | <https://omim.org/entry/603904> |
| *JAG1* | NM_000214 |  |  | <https://omim.org/entry/601920> |
| *KCNJ13* | NM_002242 |  |  | <https://omim.org/entry/603208> |
| *KCNV2* | NM_133497 |  |  | <https://omim.org/entry/607604> |
| *KIAA1549* | NM_001164665 |  |  | <https://omim.org/entry/613344> |
| *KIF11* | NM_004523 |  |  | <https://omim.org/entry/148760> |
| *KIF3B* | NM_004798 |  |  | <https://omim.org/entry/603754> |
| *KIZ* | NM_018474 |  |  | <https://omim.org/entry/615757> |
| *KLHL7* | NM_001031710 |  |  | <https://omim.org/entry/611119> |
| *LAMA1* | NM_005559 |  |  | <https://omim.org/entry/150320> |
| *LCA5* | NM_181714 |  |  | <https://omim.org/entry/611408> |
| *LRAT* | NM_004744 |  |  | <https://omim.org/entry/604863> |
| *LRIT3* | NM_198506 |  |  | <https://omim.org/entry/615004> |
| *LRP5* | NM_002335 |  |  | <https://omim.org/entry/603506> |
| *LZTFL1* | NM_020347 |  |  | <https://omim.org/entry/606568> |
| *MAK* | NM_001242957 |  |  | <https://omim.org/entry/154235> |
| *MAPKAPK3* | NM_001243926 |  |  | <https://omim.org/entry/602130> |
| *MCAT* | NM_173467 |  |  | <https://omim.org/entry/614479> |
| *MERTK* | NM_006343 |  |  | <https://omim.org/entry/604705> |
| *MFN2* | NM_014874 |  |  | <https://omim.org/entry/608507> |
| *MFRP* | NM_031433 |  |  | <https://omim.org/entry/606227> |
| *MFSD8* | NM_152778 |  |  | <https://omim.org/entry/611124> |
| *MIEF1* | NM_019008 |  |  | <https://omim.org/entry/615497> |
| *MKKS* | NM_018848 |  |  | <https://omim.org/entry/604896> |
| *MKS1* | NM_017777 |  |  | <https://omim.org/entry/609883> |
| *MTPAP* | NM_018109 |  |  | <https://omim.org/entry/613669> |
| *MTRFR* | NM_152269 |  |  | <https://omim.org/entry/613541> |
| *MVK* | NM_000431 |  |  | <https://omim.org/entry/251170> |
| *MYO7A* | NM_000260 |  |  | <https://omim.org/entry/276903> |
| *NAALADL1* | NM_005468 |  |  | <https://omim.org/entry/602640> |
| *NBAS* | NM_015909 |  |  | <https://omim.org/entry/608025> |
| *NDP* | NM_000266 |  |  | <https://omim.org/entry/300658> |
| *NDUFS2* | NM_004550 |  |  | <https://omim.org/entry/602985> |
| *NEK2* | NM_002497 |  |  | <https://omim.org/entry/604043> |
| *NEUROD1* | NM_002500 |  |  | <https://omim.org/entry/601724> |
| *NMNAT1* | NM_022787 |  |  | <https://omim.org/entry/608700> |
| *NPHP1* | NM_000272 |  |  | <https://omim.org/entry/607100> |
| *NPHP3* | NM_153240 |  |  | <https://omim.org/entry/608002> |
| *NPHP4* | NM_015102 |  |  | <https://omim.org/entry/607215> |
| *NR2E3* | NM_014249 |  |  | <https://omim.org/entry/604485> |
| *NR2F1* | NM_005654 |  |  | <https://omim.org/entry/132890> |
| *NRL* | NM_006177 |  |  | <https://omim.org/entry/162080> |
| *NYX* | NM_022567 |  |  | <https://omim.org/entry/300278> |
| *OAT* | NM_000274 |  |  | <https://omim.org/entry/613349> |
| *OFD1* | NM_003611 | c.935+706A>G | chrX: 13750239 | <https://omim.org/entry/300170> |
| *OPA1* | NM_130837 | c.610+364G>A | chr3: 193618201 | <https://omim.org/entry/605290> |
| *OPA3* | NM_025136 |  |  | <https://omim.org/entry/606580> |
| *OTX2* | NM_172337 |  |  | <https://omim.org/entry/600037> |
| *PANK2* | NM_153638 |  |  | <https://omim.org/entry/606157> |
| *PAX2* | NM_003987 |  |  | <https://omim.org/entry/167409> |
| *PCARE* | NM_001029883 |  |  | <https://omim.org/entry/613425> |
| *PCDH15* | NM_033056 |  |  | <https://omim.org/entry/605514> |
| *PCYT1A* | NM_005017 |  |  | <https://omim.org/entry/123695> |
| *PDE6A* | NM_000440 |  |  | <https://omim.org/entry/180071> |
| *PDE6B* | NM_000283 |  |  | <https://omim.org/entry/180072> |
| *PDE6C* | NM_006204 |  |  | <https://omim.org/entry/600827> |
| *PDE6G* | NM_002602 |  |  | <https://omim.org/entry/180073> |
| *PDE6H* | NM_006205 |  |  | <https://omim.org/entry/601190> |
| *PDZD7* | NM_001195263 |  |  | <https://omim.org/entry/612971> |
| *PEX1* | NM_000466 |  |  | <https://omim.org/entry/602136> |
| *PEX2* | NM_000318 |  |  | <https://omim.org/entry/170993> |
| *PEX26* | NM_017929 |  |  | <https://omim.org/entry/608666> |
| *PEX7* | NM_000288 |  |  | <https://omim.org/entry/601757> |
| *PGK1* | NM_000291 |  |  | <https://omim.org/entry/311800> |
| *PHYH* | NM_006214 |  |  | <https://omim.org/entry/602026> |
| *PITPNM3* | NM_031220 |  |  | <https://omim.org/entry/608921> |
| *PLA2G5* | NM_000929 |  |  | <https://omim.org/entry/601192> |
| *PLK4* | NM_014264 |  |  | <https://omim.org/entry/605031> |
| *PNPLA6* | NM_006702 |  |  | <https://omim.org/entry/603197> |
| *POC1B* | NM_172240 |  |  | <https://omim.org/entry/614784> |
| *POC5* | NM_001099271 |  |  | <https://omim.org/entry/617880> |
| *POMGNT1* | NM_001243766 |  |  | <https://omim.org/entry/606822> |
| *PRCD* | NM_001077620 |  |  | <https://omim.org/entry/610598> |
| *PRDM13* | NM_021620 | DNase1 hypersensitivity site | chr6:99593012-99593171 | <https://omim.org/entry/616741> |
| *PROM1* | NM_006017 | c.2077-521A>G  c.2281–26_-17del | chr4:15988237  chr4:15984372-15984381 | <https://omim.org/entry/604365> |
| *PROS1* | NM_000313 |  |  | <https://omim.org/entry/176880> |
| *PRPF3* | NM_004698 |  |  | <https://omim.org/entry/607301> |
| *PRPF31* | NM_015629 |  |  | <https://omim.org/entry/606419> |
| *PRPF4* | NM_004697 |  |  | <https://omim.org/entry/607795> |
| *PRPF6* | NM_012469 |  |  | <https://omim.org/entry/613979> |
| *PRPF8* | NM_006445 |  |  | <https://omim.org/entry/607300> |
| *PRPH2* | NM_000322 |  |  | <https://omim.org/entry/179605> |
| *PRPS1* | NM_002764 |  |  | <https://omim.org/entry/311850> |
| *RAB28* | NM_004249 |  |  | <https://omim.org/entry/612994> |
| *RAX2* | NM_032753 |  |  | <https://omim.org/entry/610362> |
| *RB1* | NM_000321 |  |  | <https://omim.org/entry/614041> |
| *RBP3* | NM_002900 |  |  | <https://omim.org/entry/180290> |
| *RBP4* | NM_006744 |  |  | <https://omim.org/entry/180250> |
| *RCBTB1* | NM_018191 |  |  | <https://omim.org/entry/607867> |
| *RD3* | NM_183059 |  |  | <https://omim.org/entry/180040> |
| *RDH11* | NM_016026 |  |  | <https://omim.org/entry/607849> |
| *RDH12* | NM_152443 |  |  | <https://omim.org/entry/608830> |
| *RDH5* | NM_002905 |  |  | <https://omim.org/entry/601617> |
| *REEP6* | NM_001329556 |  |  | <https://omim.org/entry/609346> |
| *RGR* | NM_001012720 |  |  | <https://omim.org/entry/600342> |
| *RGS9* | NM_003835 |  |  | <https://omim.org/entry/604067> |
| *RGS9BP* | NM_207391 |  |  | <https://omim.org/entry/607814> |
| *RHO* | NM_000539 |  |  | <https://omim.org/entry/180380> |
| *RIMS1* | NM_014989 |  |  | <https://omim.org/entry/606629> |
| *RLBP1* | NM_000326 |  |  | <https://omim.org/entry/180090> |
| *ROM1* | NM_000327 |  |  | <https://omim.org/entry/180721> |
| *RP1* | NM_006269 |  |  | <https://omim.org/entry/603937> |
| *RP1L1* | NM_178857 |  |  | <https://omim.org/entry/608581> |
| *RP2* | NM_006915 |  |  | <https://omim.org/entry/300757> |
| *RP9* | NM_203288 |  |  | <https://omim.org/entry/607331> |
| *RPE65* | NM_000329 |  |  | <https://omim.org/entry/180069> |
| *RPGR* | NM_001034853 |  |  | <https://omim.org/entry/312610> |
| *RPGRIP1* | NM_020366 | c.1468-263G>C c.1611+27G>A  c.2367+23delG | chr14: 21320996  chr14: 21321429  chr14: 21325406 | <https://omim.org/entry/605446> |
| *RPGRIP1L* | NM_015272 |  |  | <https://omim.org/entry/610937> |
| *RS1* | NM_000330 |  |  | <https://omim.org/entry/300839> |
| *RTN4IP1* | NM_032730 |  |  | <https://omim.org/entry/610502> |
| *SAG* | NM_000541 |  |  | <https://omim.org/entry/181031> |
| *SAMD11* | NM_152486 |  |  | <https://omim.org/entry/616765> |
| *SCAPER* | NM_020843 |  |  | <https://omim.org/entry/611611> |
| *SCLT1* | NM_144643 |  |  | <https://omim.org/entry/611399> |
| *SDCCAG8* | NM_006642 |  |  | <https://omim.org/entry/613524> |
| *SEMA4A* | NM_022367 |  |  | <https://omim.org/entry/607292> |
| *SEMA6B* | NM_032108 |  |  | <https://omim.org/entry/608873> |
| *SLC24A1* | NM_004727 |  |  | <https://omim.org/entry/603617> |
| *SLC25A46* | NM_138773 |  |  | <https://omim.org/entry/610826> |
| *SLC4A7* | NM_003615 |  |  | <https://omim.org/entry/603353> |
| *SLC7A14* | NM_020949 |  |  | <https://omim.org/entry/615720> |
| *SNRNP200* | NM_014014 |  |  | <https://omim.org/entry/601664> |
| *SPATA7* | NM_018418 |  |  | <https://omim.org/entry/609868> |
| *SPG7* | NM_003119 |  |  | <https://omim.org/entry/602783> |
| *SPP2* | NM_006944 |  |  | <https://omim.org/entry/602637> |
| *SSBP1* | NM_003143 |  |  | <https://omim.org/entry/600439> |
| *TEAD1* | NM_021961 |  |  | <https://omim.org/entry/189967> |
| *TIMM8A* | NM_004085 |  |  | <https://omim.org/entry/300356> |
| *TIMP3* | NM_000362 |  |  | <https://omim.org/entry/188826> |
| *TMEM126A* | NM_032273 |  |  | <https://omim.org/entry/612988> |
| *TMEM216* | NM_001173990 |  |  | <https://omim.org/entry/613277> |
| *TMEM237* | NM_001044385 |  |  | <https://omim.org/entry/614423> |
| *TOPORS* | NM_005802 |  |  | <https://omim.org/entry/609507> |
| *TREX1* | NM_033629 |  |  | <https://omim.org/entry/606609> |
| *TRIM32* | NM_012210 |  |  | <https://omim.org/entry/602290> |
| *TRNT1* | NM_182916 |  |  | <https://omim.org/entry/612907> |
| *TRPM1* | NM_002420 |  |  | <https://omim.org/entry/603576> |
| *TSPAN12* | NM_012338 |  |  | <https://omim.org/entry/613138> |
| *TTC8* | NM_144596 |  |  | <https://omim.org/entry/608132> |
| *TTLL5* | NM_015072 |  |  | <https://omim.org/entry/612268> |
| *TTPA* | NM_000370 |  |  | <https://omim.org/entry/600415> |
| *TUB* | NM_003320 |  |  | <https://omim.org/entry/601197> |
| *TUBGCP4* | NM_014444 |  |  | <https://omim.org/entry/609610> |
| *TUBGCP6* | NM_020461 |  |  | <https://omim.org/entry/610053> |
| *TULP1* | NM_003322 |  |  | <https://omim.org/entry/602280> |
| *UNC119* | NM_005148 |  |  | <https://omim.org/entry/604011> |
| *USH1C* | NM_153676 |  |  | <https://omim.org/entry/605242> |
| *USH1G* | NM_173477 |  |  | <https://omim.org/entry/607696> |
| *USH2A* | NM_206933 | c.5573-834A>G c.8845+628C>T  c.9959-4159A>G  c.14134-3169A>G | chr1: 216074134  chr1: 215866379  chr1: 215794441  chr1: 215653970 | <https://omim.org/entry/608400> |
| *USP45* | NM_001080481 |  |  | <https://omim.org/entry/618439> |
| *VCAN* | NM_004385 |  |  | <https://omim.org/entry/118661> |
| *WDPCP* | NM_015910 |  |  | <https://omim.org/entry/613580> |
| *WDR19* | NM_025132 |  |  | <https://omim.org/entry/608151> |
| *WFS1* | NM_006005 |  |  | <https://omim.org/entry/606201> |
| *WHRN* | NM_015404 |  |  | <https://omim.org/entry/607928> |
| *YME1L1* | NM_014263 |  |  | <https://omim.org/entry/607472> |
| *ZNF408* | NM_024741 |  |  | <https://omim.org/entry/616454> |
| *ZNF423* | NM_015069 |  |  | <https://omim.org/entry/604557> |
| *ZNF513* | NM_144631 |  |  | <https://omim.org/entry/613598> |

**Supplementary Table 3 (see Excel File). Pathogenic and likely pathogenic alleles identified in 7,017 patients with inherited retinal dystrophies.** Listed are all (likely) pathogenic variants identified in a) patients with molecular diagnosis and b) without molecular diagnosis. In solved cases hypomorphic alleles, which were classified as variants of unknown significance (VUS), were given in case they were observed in combination with a (likely) pathogenic allele in the same gene, as this combination was considered to be causative for disease. In case patients were not solved VUS were given if they were located in the same gene as a (likely) pathogenic allele. The phase of the identified variants has been classified based on the availability and examination of parental samples. When both parents were available for testing, the phase of the variants was classified as "confirmed". If only one parent or family member was available, the phase was designated as "assumed". In cases where more than two alleles were identified in a single gene and the phase could be determined or assumed from the available family data, the presumed nomenclature for these instances is indicated in the phase column. Age at genetic testing is given in years. Del/dup Exon = indicate the respective exon number in case whole exons are deleted or duplicated, Ex = Exon, cPos = coding position, pPos = protein position, het = heterozygous allele, hom = homozygous allele, hemi = hemizygous allele, LP = likely pathogenic, P = pathogenic, PMID = PubMed ID, HGMD = Human Gene Mutation Database. ACHM = Achromatopsia, BBS = Bardet-Biedl syndrome, CD = Cone dystrophy, CHM = Choroideremia, CRD = Cone rod dystrophy, CSNB = Congenital stationary night blindness, FEVR = familiar exudative vitreoretinopathy, ION = inherited optic neuropathies, LCA = Leber congenital amaurosis, MD = Macular dystrophy, Misc. = Miscellaneous, RP = Retinitis pigmentosa, USH = Usher syndrome, XLRS = X-linked retinoschisis.

**Supplementary Table 4 (see Excel File). Causative genes identified in 3,054 IRD and 211 ION cases with molecular diagnosis.** Given are all genes which were identified in solved patients. Overall, 139 genes have been identified to be disease-causing at least in one patient. In 5 patients more than one potential causative gene was identified, respectively in four patients with RP and one patient with CD/CRD. In these cases, both potential causative genes were considered. The statistics about how often each single gene was identified in the overall cohort and in each indication group are displayed.
